# The Role of Calcium Signaling in Sensorineural Hearing Loss

**DOI:** 10.7150/ijms.119492

**Published:** 2025-09-21

**Authors:** Yun Hu, Juanjuan Li, Li Tian, Peng Zhang, Xianhai Zeng

**Affiliations:** 1 Department of Graduate and Scientific Research, Zunyi Medical University Zhuhai Campus, Zhuhai, Guangdong, China.; 2 Department of Otolaryngology, Longgang Otolaryngology hospital & Shenzhen Key Laboratory of Otolaryngology, Shenzhen Institute of Otolaryngology, Shenzhen, Guangdong, China.

**Keywords:** Calcium, Sensorineural Hearing Loss, Hair Cells, Mechanotransduction, Therapy

## Abstract

Sensorineural hearing loss (SNHL)'s incidence is on the rise, severely affecting the quality of life of patients and even causing psychological and mental damage. It also poses a heavy burden on the global healthcare system. The auditory process involves the conversion of mechanical signals generated by the vibration of the basilar membrane into electrical signals by sensory hair cells. These signals are then transmitted to the spiral ganglion neurons, which receive input from inner hair cells and relay the information to the cochlear nucleus in the brainstem, and subsequently to the auditory cortex. Calcium plays a crucial role in this process, influencing homeostasis of the cochlear environment, the mechanoelectrical transduction channels and synaptic neurotransmitter release. Due to the increasing risks associated with aging, noise exposure, ototoxic drugs, and genetic mutations, the incidence of SNHL is continuously rising. Notably, SNHL often manifests as a disruption of calcium homeostasis. Therefore, it is essential to understand the potential mechanisms of calcium signaling in SNHL, providing new insights into the pathogenesis and treatment of SNHL. This review focuses on the mechanisms of calcium signaling in SNHL, including factors affecting calcium homeostasis and potential therapeutic approaches.

## Introduction

Hearing loss has become a significant global public health challenge, not only impeding language acquisition, social interaction, and emotional expression, but also severely affecting patients' career development and quality of life. Currently, in China, approximately 104 million people are affected by mild or more severe hearing impairments. It is projected that at least 700 million individuals will require hearing rehabilitation to improve their functionality by 2050^1^. Research has found that in China, approximately 1 to 3 infants out of every 1000 newborns are affected by hearing loss^2^, ^3^. SNHL refers to the impairment of sound perception or the conduction of neural impulses, caused by damage to the auditory pathways, auditory nerve and hair cells. It is the most common type of hearing impairment in clinical practice^4^. Sudden SNHL, ototoxic drug-induced hearing loss, presbycusis, genetic hearing loss, noise-induced hearing loss are most common types of SNHL. In the sensory conduction process of the inner ear, hair cells convert the mechanical signals of sound into electrical signals, which are then transmitted to the central nervous system. This process involves a variety of physiological mechanisms.

Ca^2+^ are key second messengers involved in cellular signaling^5^. Studies have shown that in the inner ear, Ca^2+^ are involved in a variety of physiological processes, including signal transduction in cochlear hair cells, homeostasis of the cochlear environment, and neurotransmitter release^6^, ^7^. Abnormal concentrations of Ca^2+^ in the endolymph and perilymph can impair normal auditory function. Therefore, maintaining a relatively stable concentration of Ca^2+^ in the endolymph and perilymph is crucial for auditory function. This review focuses on the central role of calcium signaling in auditory signal transduction and systematically elaborates on its regulatory mechanisms in mechanotransduction and neurotransmitter release. The first part dissects the pathological associations between dysfunction of key molecules in calcium homeostasis (such as ion channels, calcium pumps, and mitochondrial uniporters) and SNHL. The second part evaluates the translational potential and future directions of calcium signaling-targeted intervention strategies in the treatment of deafness, attempting to propose innovative strategies for the prevention and treatment of SNHL.

## The Physiological Role of Calcium in Hearing

**Figure 1:**
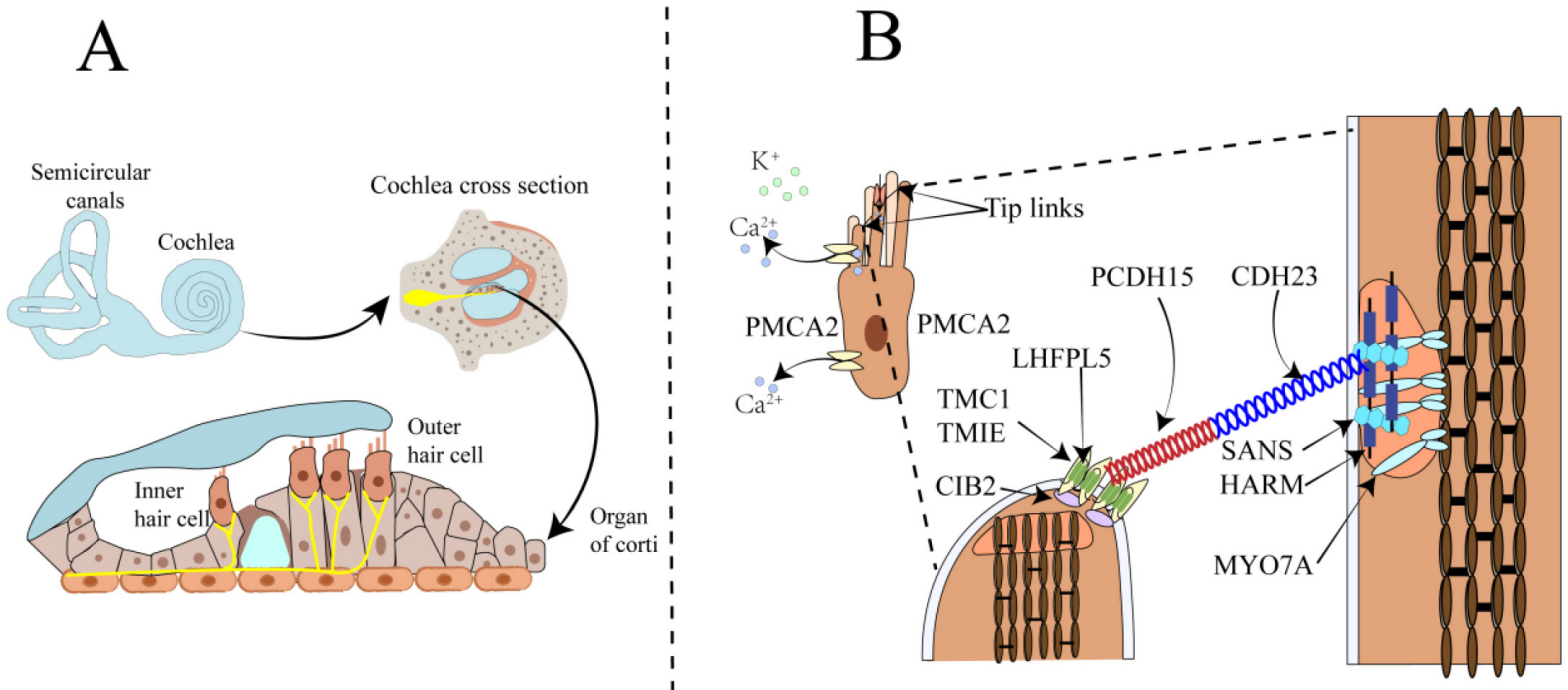
** Schematic diagram of the anatomy of the mammalian cochlea, hair cells, and the MET apparatus. A.** The upper part shows the cochlea and the semicircular canals responsible for balance control. The lower part illustrates the three rows of outer hair cells OHCs and one row of IHCs within the organ of Corti in the cochlea. OHCs amplify the incoming sound signals and are connected to efferent neurons. IHCs serve as the primary sensory receptor cells, innervated by afferent nerve fibers that transmit sound information to the central nervous system. **B.** Illustrates the composition of the tip-link and the MET channel. The MET channel is primarily composed of TMC1 and TMC2, TMIE, LHFPL5, and CIB2. When hair cells are mechanically stimulated, the shorter stereocilia deflect toward the taller stereocilia. This deflection generates tension in the tip-link, which opens the MET channel located at the tip of the shorter stereocilium. As a result, Ca²⁺ and K⁺ ions flow into the hair cell, causing depolarization of the hair cell.

The cochlea, resembling a snail shell in shape and having a spiral structure, is divided into three chambers by the vestibular membrane and the basilar membrane. The scala vestibuli and scala tympani are filled with perilymph, while the scala media is filled with endolymph. The cochlea is composed of different structures, including the three chambers, the basilar membrane, the organ of Corti, the stria vascularis, the spiral ligament, the bony spiral lamina, and the auditory nerve. The organ of Corti consists of a single row of inner hair cells and three rows of outer hair cells, along with various supporting cells (Figure 1A). It is responsible for sound conduction, located on the basilar membrane, and bathed in endolymph. The endolymph in the inner ear is a unique extracellular fluid characterized by low Na⁺ and low Ca²⁺ but high K⁺^8^-^11^. Notably, the Ca^2+^ concentration in endolymph is just 20 μM, significantly lower than the 1-2 mM found in perilymph and other extracellular fluids^6^.

Sound wave vibrations transmitted to the cochlea via the oval window induce a shearing motion in both the basilar and tectorial membranes. The deflection of stereocilia at the apex of hair cells concurrently leads to the further opening of mechanosensitive channels at their tips. The stereocilia are rigid microvilli composed of 20 to 300 actin filaments, arranged in a staircase-like pattern. Cadherin-23 (CDH23) and protocadherin-15 (PCDH15) are calcium-dependent transmembrane adhesion proteins that constitute the upper and lower segments of the tip-link, respectively^12^-^14^. The homodimers of PCDH15 interact in an antiparallel manner with the homodimers of CDH23, and this interaction is calcium-dependent. Kinocilia are cilia filled with microtubules, present in developing hair cells but absent in mature hair cells of the cochlea. Studies have shown that the MET channels are located at the tips of the shorter stereocilia at the lower end of the tip-link^15^-^17^. The tip-link extends from the tip of a shorter stereocilium to the side wall of an adjacent taller stereocilium^18^-^20^. The tension of the tip-link is considered to be transmitted to the MET channel via a gating spring connected to the tip-link^21^. Sound-induced vibrations cause shorter stereocilia to deflect towards the tallest adjacent stereocilium, increasing tip-link tension and opening the MET channel^22^. Reducing extracellular Ca^2+^ to sub-micromolar levels using the Ca^2+^ chelator BAPTA causes tip-link disassembly and eliminates transduction^7^. In the mammalian cochlea, sensory hair cells are responsible for sound conduction, and inner hair cells (IHCs) and outer hair cells (OHCs) have distinct functions in the auditory process. OHCs amplify the incoming sound signals and are connected to efferent neurons, playing a crucial role in signal amplification (Figure 1B). In contrast, IHCs are innervated by afferent nerve fibers that transmit auditory information to the central nervous system^7^, ^23^. The complex physiological process of auditory perception, from sound detection to neural signal transmission, requires the coordinated action of both inner and outer hair cells.

## The Impact of Calcium Channels Dysfunction in Hearing Loss

### Voltage-gated channels

Voltage-gated channels play important roles in auditory perception and information processing in the inner ear and brainstem. Voltage-gated channels include high-voltage-activated calcium channels and low-voltage-activated calcium channels. High-voltage-activated calcium channels include L-type (Cav1.1, Cav1.2, Cav1.3, and Cav1.4), P/Q-type (Cav2.1), N-type (Cav2.2), and R-type (Cav2.3), which require significant membrane depolarization to open. Low-voltage-activated calcium channels, also known as T-type Ca^2+^ channels, can be divided into three subtypes based on their encoding genes: Cav3.1, Cav3.2, and Cav3.3. These are encoded by the CACNA1G, CACNA1H, and CACNA1I genes, respectively. They can be activated by small voltage changes near the typical resting membrane potential of neurons^24^-^26^. The calcium channel primarily associated with hearing is the Cav1.3 L-type calcium channel. Knockout of this channel leads to congenital deafness. Subsequent studies have found that the Cav3.2 T-type calcium channel may play a role in age-related and noise-induced hearing loss.

#### CaV1 (L-type)

In mammalian inner and outer hair cells, the current generated by the Cav3.1 channel accounts for over 90% of the total hair cell current. Therefore, dysfunction of the Cav3.1 calcium channel can lead to hearing loss, with deafness being explained by the near-complete loss of Ca²⁺ influx^27^, ^28^. The CaV1.3 calcium channel is the principal voltage-gated calcium channel in the inner ear, coupling Ca²⁺ influx and neurotransmitter release in inner hair cells of the cochlea to sound-induced membrane potential changes. The central auditory system's development is crucial.

The CaV1.3 calcium channel is essential for cochlear development, maintaining cochlear homeostasis, and neurotransmitter release, all critical for hearing^27^, ^29^, ^30^. Research indicates that CaV1.3 gene knockout mice experience congenital deafness due to the significant lack of L-type calcium channels in hair cells, resulting in auditory hair cell degeneration^27^, ^29^, ^31^. It has been reported that mutations in the CACNA1D gene, which encodes the Cav1.3 α1 subunit, lead to a human disease known as sinoatrial node dysfunction and deafness syndrome. This syndrome is primarily characterized by congenital deafness and severe sinoatrial node dysfunction. It mainly occurs in consanguineous deaf families, and the human phenotype of deafness and sinoatrial node dysfunction is very similar to that of mice with complete knockout of the Cav1.3 channel^30^, ^32^.

The Cav1.3 may be associated with age-related hearing loss (ARHL) and noise-induced hearing loss (NIHL). Understanding their involvement in the pathogenesis of hearing loss also identifies them as potential targets for prevention and treatment. The gradual decrease in Cav1.3 expression in the stria vascularis, IHCs and OHCs with increasing age suggests a potential role of Cav1.3 in ARHL^33^.

#### CaV3 (T-type)

T-type Ca²⁺ channels, also known as voltage-dependent calcium channels, are characterized by rapid activation, rapid inactivation, and low-voltage activation (at around -60 mV). Within the Cav3 family of voltage-gated calcium channels, the Cav3.2 channel is the predominant subtype in the cochlea. Mutations in the CACNA1H gene, which encodes the Cav3.2 T-type calcium channel, are risk factors for many human channelopathies, including epilepsy^34^, retinal dysfunction^35^, and hearing loss. Cav3.2 plays a vital role in young individuals. However, in cases of age-related or noise-induced acquired sensorineural hearing loss, an increase in Cav3.2 expression levels may have detrimental effects on the survival of hair cells and spiral ganglion neurons (SGNs). Knocking out Cav3.2 in mice can delay age-related cochlear functional decline and damage to SGNs, further highlighting the potential role of Cav3.2 in ARHL^36^. Currently, research on the expression characteristics of Cav3.2 in the mouse cochlea remains relatively limited, and its functional role in the human auditory system has not yet been clearly elucidated. Meanwhile, although Cav3.1 and Cav3.3 are expressed at low levels in the cochlea, their potential contributions to hearing loss still need to be further explored.

### Other calcium channels

During the mechanotransduction process of inner ear hair cells, several ion channels have been proposed as candidate components and have been widely studied. Members of the Transient Receptor Potential (TRP) channel family are believed to be potentially involved in this process. For example, in zebrafish, TRPN1 has been proven to play a key role in mechanosensitivity. Knockdown of TRPN1 not only leads to the loss of FM1-43 dye uptake and the disappearance of microphonic potentials but also causes deafness, indicating that TRPN1 has an important function in the mechanotransduction of non-mammalian hair cells^37^. In mammals, TRPA1 has been found to be expressed at the apical pole of cochlear hair cells and is thought to be involved in mechanotransduction, but there is still a lack of clear structural and functional evidence to support its core role^38^.

On the other hand, Piezo1 and Piezo2 are the more extensively studied mechanosensitive ion channels, Piezo1 and Piezo2 are evolutionarily conserved multi-transmembrane domain proteins in vertebrates. As core components of mechanically-activated (MA) cation channels, they directly mediate mechanically gated cation influx and play essential roles in various physiological processes. Although Piezo2 is expressed in OHCs and vestibular hair cells, it does not constitute the primary sensory transduction channel in the stereocilia. A study by Wu *et al.* showed that Piezo2 is located on the apical surface of OHCs and can mediate the reverse polarity mechanosensitive current that appears when tip links are damaged or the transduction mechanism fails. This suggests that there may be two types of mechanotransduction channels in hair cells with different functions and molecular compositions: one type formed by TMC1/TMC2, which is responsible for normal mechanosensation, and the other involving Piezo2, which is responsible for the reverse current generated under specific conditions. It is worth noting that the response mediated by Piezo2 is regulated by intracellular Ca²⁺ concentration and may play a compensatory or regulatory role during early development or after mechanical injury^39^.

In addition, the core molecules of the Store-Operated Calcium Entry system, Orai1 and STIM1, are widely present in non-excitable cells and regulate Ca²⁺ influx. There is currently no evidence to suggest that they are directly involved in the mechanotransduction or auditory perception mechanisms of hair cells. However, they may play a certain auxiliary role in maintaining calcium homeostasis in cochlear cells.

## Connexins and Calcium Pumps in Hearing Loss

### Plasma membrane calcium ATPase

The Plasma Membrane Ca²⁺-ATPase (PMCA) is a core family of proteins that maintain intracellular calcium homeostasis by actively expelling Ca²⁺ from the cytoplasm to the extracellular space, consuming ATP in the process. This action prevents the cytotoxic effects caused by calcium overload. The PMCA family consists of four subtypes (PMCA1-4), which are encoded by different genes (ATP2B1-ATP2B4). These subtypes exhibit significant tissue-specificity in their distribution and function. The PMCA1 and PMCA4 subtypes are widely expressed in almost all tissues, whereas PMCA2 and PMCA3 are expressed in specific tissues, primarily in cells of brain and neural origin^40^-^42^. Maintaining Ca²⁺ homeostasis is crucial for auditory function. During auditory signal transduction, the mechanical motion of hair cells activates the MET channels, causing Ca²⁺ influx and triggering neurotransmitter release. PMCA2 rapidly clears Ca²⁺ from within the stereocilia, maintaining a low-calcium microenvironment that ensures the efficient cycling of mechanotransduction channel opening and closing, thereby ensuring precise auditory signal transmission. PMCA2 is highly expressed in the hair bundles and apical surfaces of vestibular hair cells and outer hair cells, while its expression is lower in inner hair cells^43^, ^44^. PMCA2 has distinct characteristics compared to the other three PMCA subtypes. It has a higher affinity for calmodulin (CaM) and can pump Ca²⁺ out of the cell at a relatively high constant rate even in the absence of CaM.

PMCA2 and the plasma membrane Na⁺/Ca²⁺ exchanger (NCX) are the primary mechanisms for intracellular Ca²⁺ clearance and play crucial roles in the regulation of Ca²⁺ homeostasis. PMCA2 has a high affinity for Ca²⁺ and can be rapidly activated when Ca²⁺ levels rise, although its transport capacity is low. In contrast, NCX has a lower affinity for Ca²⁺ but a higher transport capacity, relying on the Na⁺ concentration gradient to facilitate Ca²⁺ exchange. However, the concentration of Na⁺ in the endolymph is extremely low. Therefore, in the context of rapid and sustained Ca²⁺ clearance in hair cells, PMCA2 is more critical. Studies have shown that Ca²⁺ extrusion in hair cells is mediated by the PMCA2 pump^45^, ^46^. So far, no studies have demonstrated the presence of Na⁺/Ca²⁺ exchangers on hair cells. PMCA2 can selectively insert three exons of 33, 60, and 43 nucleotides at splice site A. These variants include the w variant (containing all three exons), the z variant (with no exon insertion), the x variant (containing only the 42nt exon), and the y variant (including both the 33nt and 60nt exons)^42^, ^47^. At splice site C, two exons of 172 and 55 nucleotides can be selectively inserted. These variants include variant a (containing both exons), variant b (with no exon insertion), and variant c (containing only the 172nt exon). The insertion of these exons at this site leads to a truncation approximately 50 residues upstream of the original C-terminus, occurring within the binding domain for CaM. Even in the absence of CaM, PMCA2 retains the ability to efficiently pump out Ca²⁺^42^. The double-splice variant w/a of PMCA2 is predominantly located in stereocilia. Ablation or missense mutations in the PMCA2 Ca²⁺ pump leads to deafness phenotypes, and Ca²⁺ extrusion is also impaired, resulting in reduced Ca²⁺ concentration in the endolymph. Mutations in the PMCA2 (A2b2) gene affect the deafness phenotypes in mice and humans as well as the function of the protein (Table 1). Therefore, mutations in PMCA2 enhance the deafness phenotype caused by cadherin-23 mutations, leading to severe hearing loss, and the two exhibit a synergistic effect^48^.

PMCA2, as a key calcium pump maintaining calcium homeostasis in cochlear hair cells, is closely associated with various sensorineural deafness mouse models. Multiple studies using mouse models (such as deafwaddler, Tommy, and Oblivion mutations) have revealed a direct link between PMCA2 dysfunction and hearing loss. Street *et al.* first reported that the deafwaddler (dfw) mutation is a point mutation (G283S) in the Atp2b2 gene, which significantly inhibits the calcium-pumping function of PMCA2, leading to auditory and vestibular dysfunction in mice^49^. Subsequently, Takahashi and Kitamura *et al.* confirmed that this point mutation leads to the loss of PMCA2 pump activity, suggesting that mutations in PMCA2 may be a potential pathogenic factor^50^. The PMCA2 knockout mouse model revealed that the loss of calcium pump function leads to calcium homeostasis imbalance in hair cell stereocilia and hair cell degeneration, thereby causing severe sensorineural hearing loss and balance disorders^51^. Subsequent studies identified the Atp2b2 mutant allele dfwi5, and its homozygous mutant mice also exhibited complete deafness, severe vestibular motor disorders, and disordered hair cell cilia structure^52^. In dfw2J mice, a deletion mutation in Atp2b2 results in a complete lack of PMCA2 protein expression, leading to more severe hearing and vestibular phenotypes. The Ca²⁺ concentration in the endolymph of their cochlea is significantly reduced, indicating the crucial role of PMCA2 in maintaining endolymphatic Ca²⁺ levels^53^. The Oblivion mutation (N1110K) causes abnormal membrane localization and loss of function of PMCA2. The mouse model of this mutation exhibits typical hearing defects and vestibular disorders, indicating that the mutation causes disease by affecting the structural stability of PMCA2^54^. The Tommy mutation (D564N) disrupts the calcium clearance efficiency of PMCA2, leading to residual calcium accumulation within hair cells. This, in turn, causes cellular dysfunction and results in hearing loss^55^. In the complete knockout model of the Atp2b2 gene, the absence of PMCA2 leads to significant vestibular dysfunction and severe deafness, further supporting the critical role of PMCA2 in the normal function of hair cells^56^. Additionally, Tasi *et al.* identified a *de novo* mutation originating from embryonic stem cells that also led to similar phenotypes, suggesting the potential risk of spontaneous mutations in the Atp2b2 gene^57^. Ficarella *et al.* conducted *in vitro* functional studies on various PMCA2 mutants and found that different mutations can collectively cause PMCA2 dysfunction through multiple mechanisms, such as reduced enzyme activity and altered membrane localization. This further elucidates the deafness-causing mechanisms at the molecular level^58^.

In summary, although different types of Atp2b2 mutations vary in their molecular mechanisms and phenotypes, they all disrupt the function of PMCA2, thereby affecting calcium homeostasis in hair cells and leading to sensorineural hearing loss. This is an important molecular basis for hereditary deafness.

### Calcium and integrin-binding protein 2

Mutations in the Calcium and integrin-binding protein 2 (CIB2) gene disrupt calcium signaling regulation and integrin interactions, leading to dysfunction of inner ear hair cells and causing non-syndromic deafness (DFNB48) and Usher syndrome (USH1J). CIB2 is primarily located in the stereocilia of the inner ear and mainly interacts with myosin VIIa and whirlin^59^,highlighting the central role of calcium homeostasis in the auditory system.

After the pathogenicity of USH1J was established, researchers identified various pathogenic variants of the CIB2 gene in families presenting with DFNB48-type isolated deafness phenotype (without clinical features of retinitis pigmentosa or vestibular dysfunction)^60^-^63^. Studies have shown that CIB2 can form a complex with Transmembrane channel - like proteins (TMC) 1 and 2, participating in the regulation of mechanotransduction channel activation, current conduction, and calcium homeostasis maintenance. The absence of CIB2 leads to a complete loss of transduction current and causes severe hearing impairment^64^, ^65^. In CIB2-deficient mouse models, the development of hair cells is not disrupted, but their ability to transduce sound stimuli is severely impaired. This indicates that CIB2 primarily mediates the functional transduction of auditory signals rather than the formation of cellular structures^65^, ^66^. Further structural studies have found that CIB2 can form stable heterocomplexes with TMC family proteins and has a certain degree of functional complementarity with CIB3. It has been found that CIB3 can partially compensate for the function of CIB2 in the vestibular system. This explains why patients with CIB2 mutations mainly exhibit deafness rather than vestibular disorders, providing new insights into the study of functional compensation mechanisms^67^. In human genetic studies, it has been clearly established that mutations in CIB2 cause only DFNB48-type deafness and do not affect visual function. This finding corrects the earlier misjudgment of its association with USH1J^61^. As a core molecule of the MET channel, genetic mutations in CIB2 disrupt ion channel function, leading to depolarization defects in hair cells. This is one of the important pathogenic mechanisms of sensorineural hearing loss. It not only deepens the understanding of the MET channel complex but also provides an important basis for the study of deafness mechanisms and the development of therapeutic strategies.

### CDH23 and PCDH15

CDH23 and PCDH15 have long extracellular domains and multiple extracellular cadherin repeats. They form the upper and lower parts of the tip-link and are members of the cadherin family, together constituting the core components of the tip-link^13^, ^68^. This connection not only provides mechanical support but also directly participates in the transduction of auditory signals. Mutations in their genes are closely related to several forms of non-syndromic deafness (such as DFNB12 and DFNB23) and Usher syndrome type I (USH1).

In early studies of USH1, it was found that pathogenic mutations in CDH23 and PCDH15 are closely related to deafness and retinitis pigmentosa^68^. Mutations in the CDH23 gene can cause Usher syndrome type 1D (USH1D) and non-syndromic deafness DFNB12. Loss-of-function mutations are often associated with the typical USH1 phenotype, while missense mutations that retain partial function are more commonly seen in DFNB12, showing a clear genotype-phenotype correlation^69^, ^70^. Similarly, mutations in PCDH15 have also been confirmed to be associated with USH1F and non-syndromic deafness DFNB23. Truncating mutations often lead to typical USH1, while mutations with minor structural changes result in non-syndromic deafness^71^, ^72^. This trend is also confirmed in animal models. For example, the CDH23 mutation in waltzer mice manifests as hearing and vestibular function loss, while the V2360E missense mutation carried by jera mice only results in deafness, supporting its role as a DFNB12 model^73^. The function of PCDH15 has also been deeply understood through the Ames waltzer and noddy mouse models, in which domain deletions or key amino acid substitutions directly affect the formation and function of the tip-link^74^-^76^. Structural and functional studies have shown that the multiple extracellular cadherin domains of the CDH23 protein are rich in calcium-binding sites, and mutations in these domains can significantly affect protein stability and force-transduction capabilities^77^.

Recent studies have focused on the impact of alternative splicing regulation of CDH23 on its function. Liu *et al.* developed a mouse model lacking exon 68 and found that while this exon does not affect tip-link formation, its absence weakens the stability of the tip-link, leading to progressive and noise-induced hearing loss. Mechanistically, the peptide encoded by this exon can regulate the formation of phase-separated condensates between CDH23 and Harmonin protein, revealing its crucial role in maintaining the structural integrity of the upper tip-link density^78^. In summary, the study of deafness mechanisms caused by mutations in CDH23 and PCDH15 has progressed from the identification of functional mutations to in-depth investigations of structure-function relationships. This has provided a solid theoretical foundation for understanding the etiology of sensorineural hearing loss and developing targeted therapies^79^.

### TMCI/2

TMC1 and TMC2 play a crucial role in the mechanoelectrical transduction of hair cells and may also mediate hair cell damage and apoptosis, leading to deafness, by affecting intracellular Ca²⁺ homeostasis. TMC1 and TMC2 were initially identified in human deafness patients, and studies have shown that they also play key roles in mice and zebrafish^80^-^82^. TMC1 and TMC2 share a certain degree of homology in their molecular sequences, but they exhibit complementary expression in terms of timing and location within the inner ear: TMC2 plays a dominant role during early development, and as the auditory system matures, TMC1 gradually replaces TMC2 as the primary component^80^, ^83^.

Mutations in TMC1 have a significant impact on the permeability to Ca²⁺ and can cause autosomal dominant (DFNA36) or recessive (DFNB7/B11) non-syndromic deafness^80^. Studies have shown that the TMC1 D569N mutation (corresponding to D572N in humans) in mice reduces the permeability of the MET channel to Ca²⁺ by about threefold. This leads to weakened electrophysiological function of hair cells and cell apoptosis, ultimately resulting in early and severe hearing loss^84^. However, another mutation in TMC1, the Beethoven (Bth) mutation (M412K), also affects Ca²⁺ influx but has milder effects on the structure of hair cells and the amplitude of currents, mainly manifesting as progressive hearing loss, similar to human DFNA36^81^, ^85^. Studies have shown that the role of TMC2 deficiency in deafness should not be overlooked. It plays a crucial role in the development of hair cells, especially in the early stages. It has been found that TMC1 knockout mice exhibit complete deafness but retain vestibular function, partly because TMC2 continues to be expressed in vestibular hair cells. Interestingly, under conditions of TMC1 deficiency, transgenic expression of TMC2 can partially restore auditory function, but this compensatory effect weakens as the animal ages^86^. Moreover, the triple mutation of TMC1/2a/2b in zebrafish leads to complete hearing loss, but the morphology of hair cells remains normal and the localization of other MET components (such as PCDH15a) is unaffected^82^. TMC1 and TMC2 are key proteins in hair cells for sensing sound signals. Mutations that disrupt channel function can lead to deafness. They play a central role in the formation and maintenance of hearing and are important targets for the study and treatment of hereditary deafness.

### Connexin 26

Connexin 26 (Cx26) is widely expressed in the non-sensory epithelium of the cochlea and plays an important role in the acquisition and maintenance of hearing. Mutations in Cx26 can not only cause congenital deafness but also lead to late-onset hearing loss. In recent years, it has been discovered that Cx26 mutations related to calcium regulation can disrupt channel function through various mechanisms, resulting in hearing impairment.

Over two decades ago, Kelsell *et al.* identified the gene Gap junction protein beat 2 (GJB2), which encodes the connexin 26 (Cx26) gap junction protein, as a susceptibility gene for sensorineural hearing loss^87^. The GJB2 gene is mostly associated with recessive mutations leading to non-syndromic deafness, while a smaller portion involves dominant missense mutations causing syndromic deafness, such as the keratitis-ichthyosis-deafness (KID) syndrome. In 2010, Sanchez *et al.* discovered that the Cx26 protein hemichannels formed after the p.Gly45Glu mutation abnormally open under normal extracellular calcium concentrations. This leads to increased membrane permeability, enhanced calcium ion influx, and ultimately induces cell apoptosis. High concentrations of calcium can significantly reduce the channel activity of this mutant protein, thereby delaying cell death^88^. Similarly, the p.Gly12Arg (G12R) mutation disrupts voltage-gated channels, leading to persistent Ca²⁺ influx, resulting in calcium overload and cytotoxicity^89^. In addition, studies have found that mutations such as A40V and T55N affect the permeability of Ca²⁺ to varying degrees. The A40V mutation indirectly increases Ca²⁺ permeability by disrupting the normal closed state of the channel^90^. These mutations collectively reveal the crucial role of GJB2 in maintaining calcium homeostasis. Mutations disrupt Ca²⁺ homeostasis, leading to hearing loss.

## Otoferlin is Essential for Synaptic Transmission

Otoferlin, as one of the members of the human ferlin protein family, is a calcium sensor with six C2 domains^91^, ^92^. In mammalian IHCs, otoferlin is a key Ca^2+^ sensor for synaptic vesicle release, playing a crucial role in synaptic transmission in inner hair cells. Defects in otoferlin can lead to DFNB9 type non-syndromic deafness, a mutation typically characterized by severe hearing loss appearing in early childhood. Yasunaga *et al.* first established the direct link between OTOF gene mutations and deafness through genomic analysis of human DFNB9 deafness patients, providing the foundation for understanding the role of OTOF in auditory function^93^. Roux *et al.* further demonstrated that otoferlin is an essential protein for synaptic vesicle release in inner hair cells by studying otoferlin knockout mice. Their findings showed that loss of the OTOF gene leads to a complete arrest of synaptic vesicle exocytosis in inner hair cells, ultimately resulting in hearing loss. This study underscores the critical role of otoferlin in maintaining proper synaptic function in the inner ear^94^. In addition, Michalski *et al.* demonstrated, using a mouse model, the critical role of the C2 domain of otoferlin in synaptic vesicle fusion and pool regeneration in the inner ear. Their study further elucidated the function of otoferlin in the processes of vesicle docking and release^91^. The study by Chen *et al.* found that mutations in the C2F domain severely impair vesicle fusion, leading to hearing loss, further validating the essential role of the C2 domain of otoferlin in synaptic transmission^95^.

Although previous studies have shown that the C2 domain of otoferlin plays a key role in synaptic vesicle fusion and regeneration, the function of its transmembrane domain remains unclear. To address this, Tertrais *et al.* repaired the OTOF gene knockout mice through gene therapy and proposed that otoferlin has a dual function in inner hair cell synaptic transmission: acting as a Ca²⁺ sensor and participating in calcium channel clustering, thus promoting the recruitment of synaptic vesicles^96^. Further studies using GFP-tagged OTOF gene knock-in mice investigated the regulatory role of otoferlin's C-terminal tail in vesicle function. The results showed that although the GFP tag did not affect otoferlin's localization and expression, in homozygous GFP mice, synaptic vesicle fusion was significantly impaired, manifested by a decrease in vesicle docking ability and a reduction in fusion rate^97^. These studies provide deeper insights into the complex role of otoferlin in synaptic transmission, highlighting the critical role of its C2 domain and transmembrane domain remains in regulation.

These studies collectively highlight the indispensable role of otoferlin in synaptic vesicle release in inner hair cells, particularly the critical function of its C-terminal region in maintaining vesicle docking and fusion. These findings not only deepen our understanding of the dynamic mechanisms of synaptic vesicles but also provide valuable insights for future gene therapy strategies.

## Transporters and Calcium-Binding Proteins Regulate Calcium Homeostasis in Hearing Loss

Mitochondria play a crucial buffering role in intracellular Ca²⁺ homeostasis. The mitochondrial calcium uniporter (MCU) rapidly takes up Ca²⁺ when cytoplasmic calcium concentrations rise, reducing the rapid increase of intracellular Ca²⁺ and accelerating its clearance, thereby effectively preventing cytotoxic reactions caused by Ca²⁺ overload^98^-^100^. The release of Ca²⁺ primarily relies on two exchange mechanisms: the Ca²⁺/H⁺ exchanger and the Ca²⁺/Na⁺ exchanger. In the auditory system, mitochondria, as the center for cellular energy metabolism and a hub for regulating oxidative stress, play a key role in maintaining the excitability of cochlear hair cells, synaptic transmission, regulation of MET currents, and cell survival^99^, ^101^.

Reactive oxygen specie (ROS)-mediated apoptosis and necrosis are the core mechanisms underlying sensorineural hearing loss. Impaired mitochondrial function diminishes calcium signaling regulation, restricts energy metabolism, and increases ROS production in the cochlea, which worsens mitochondrial damage and induces cell apoptosis^102^. Research indicates that intense noise causes a significant increase in Ca^2+^ influx into hair cell mitochondria, accompanied by increased MCU expression and decreased the sodium-calcium exchanger (NCLX) expression. This leads to mitochondrial calcium overload, which initiates the opening of the permeability transition pore. This subsequently causes mitochondrial membrane potential loss, ROS accumulation, and cell apoptosis, leading to damage in outer hair cells and synapses^103^. Moreover, Ca²⁺ overload can also activate the Calcium/calmodulin-dependent protein kinase kinase β-AMP-activated protein kinase alpha subunit (CaMKKβ-AMPKα) pathway, disrupting mitochondrial energy metabolism and exacerbating hair cell damage. Inhibition of mitochondrial calcium uptake or RNA interference targeting CaMKKβ can effectively reduce noise-induced hair cell loss and hearing loss^104^.

Mitochondrial DNA (mtDNA) is highly prone to accumulate mutations in metabolically active tissues due to its lack of an effective repair mechanism. Kim and other researchers found that in mitochondrial mutator mice (Polg^mut/mut^) mice, a significant increase in the number of mtDNA point mutations and deletions is closely associated with early-onset hearing loss^105^. These mutations mainly lead to the degeneration of SGNs rather than the direct death of hair cells, indicating that mtDNA damage induces age-related hearing loss (AHL) through neural output disorders. Studies have found that mutations in the mitochondrial-localized regulatory protein Fus1 can trigger mitochondrial dysfunction and a decrease in antioxidant capacity, leading to the degeneration of the cochlear microvasculature and a decline in endolymphatic potential, thus promoting age-related hearing loss^106^. In addition, mtDNA mutations continuously accumulate during the normal aging process, and this mechanism has also been observed in the pathology of human AHL. The study by Kwon *et al.* showed that the downregulation of sirtuin 3 expression will weaken the expression of antioxidant enzymes (such as MnSOD and Foxo1), leading to a disorder in ROS metabolism and the degeneration of inner ear structures, such as the loss of hair cells and the degeneration of the stria vascularis^107^. The abnormal regulation of mitochondrial proteins not only affects cellular metabolism but may also indirectly damage the function of hair cells by influencing the cochlear microenvironment.

Ca²⁺ are important factors in the regulation of mitochondrial metabolism. In terms of drug toxicity, Clinically common aminoglycoside antibiotics and platinum-based anticancer drugs can easily cause hearing loss. Recent studies have shown that these drugs mediate hair cell damage through various mechanisms, among which the disruption of mitochondrial calcium homeostasis is a key factor. Aminoglycosides can enter cells through MET channels located on the stereocilia of hair cells, leading to an increase in intracellular calcium ion concentration. The calcium ions then enter the mitochondrial matrix through the MCU. Calcium overload results in the production of ROS, which further activates the mitochondrial apoptotic pathway, ultimately leading to hair cell apoptosis^108^-^110^. Lee *et al.* discovered through a zebrafish model that cisplatin treatment induces hyperpolarization of the mitochondrial membrane potential and calcium ion overload, triggering the massive generation of ROS and apoptosis, thus disrupting the structure and function of hair cells^111^. Esterberg and others revealed that aminoglycoside drugs induce the calcium flow from the endoplasmic reticulum to the mitochondria, leading to mitochondrial Ca²⁺ overload and a ROS burst, ultimately triggering the cell death pathway. Therefore, targeting and inhibiting mitochondrial oxidation can effectively reduce the ototoxic damage caused by aminoglycoside drugs^112^, ^113^. In terms of environmental factors, the study by Wang *et al.* demonstrated that in noise-induced hearing loss, noise exposure promotes excessive mitochondrial calcium accumulation through the MCU, leading to calcium overload in cochlear hair cells, which in turn causes hair cell death and hearing impairment. The study also showed that the use of MCU inhibitors can effectively reduce noise-induced hair cell and synaptic damage^103^. Similarly, in the research on hereditary hearing impairment, Manikandan *et al.* used a mouse model with the MCU knocked out, which led to a disorder in mitochondrial calcium uptake, disrupted the calcium buffering and ATP generation systems. The mitochondrial calcium channel MCU is crucial for the Ca²⁺ buffering in cochlear hair cells. A deficiency in the MCU gene can result in the degeneration of hair cells and progressive hearing loss^114^.

In conclusion, whether it is hearing impairment caused by aging, drugs, or gene mutations, mitochondrial dysfunction is involved throughout the pathogenesis process, encompassing multiple aspects such as energy metabolism imbalance, ROS accumulation, disruption of Ca²⁺ homeostasis, and activation of apoptosis signals. Aiming at these mechanisms, future prevention and intervention strategies for deafness should focus on maintaining mitochondrial stability, enhancing antioxidant capacity, and regulating the dynamics of calcium ions, so as to achieve early protection and long-term maintenance of the auditory system.

## Calcium Signaling-Related Therapeutic Targets

### Calcium channel blockers

Calcium channel blockers have received widespread attention in the prevention and treatment of SNHL. Ca²⁺ enter the inner ear hair cells through VGCC. A moderate influx of Ca²⁺ helps maintain normal cellular function, while excessive calcium can activate a variety of harmful pathways, such as abnormal enzymatic reactions and mitochondrial damage, ultimately leading to cell apoptosis. Therefore, calcium channel blockers may have protective and therapeutic effects on SNHL.

Uemaetomari *et al.* found that pre-treatment with L-type calcium channel blockers (such as diltiazem, verapamil, nifedipine, and nimodipine) significantly reduced the elevation of auditory brainstem response thresholds and decreased hair cell loss in mice exposed to 128 dB SPL noise^115^. Heinrich *et al.* further confirmed in guinea pig experiments that the combined administration of diltiazem (75 mg/kg) before and after noise exposure has a protective effect on outer hair cells in the cochlea. This protective effect is achieved by inhibiting L-type calcium channels and reducing noise-induced Ca²⁺ overload. The study was the first to confirm the protective effects of calcium channel blockers on the cochlea at the ultrastructural level^116^. However, not all studies have shown that L-type calcium channel blockers are effective against NIHL. Boettcher *et al.* investigated the effects of diltiazem and nimodipine in a chinchilla model and found that neither the administration of 30 mg/kg diltiazem for three consecutive days nor continuous dosing during a 90 dB noise exposure improved temporary threshold shift or permanent threshold shift^117^. Similarly, Kansu *et al.* found that a single intraperitoneal injection of 3 mg/kg nimodipine in guinea pigs failed to provide protection against NIHL. These results suggest that under certain experimental conditions, L-type calcium channel blockers may be ineffective against NIHL^118^.

In contrast, T-type calcium channel blockers have shown more significant protective effects in some studies. Shen *et al.* found in the C57BL/6 mouse model that the antiepileptic drugs trimethadione (200 mg/kg/day) and ethosuximide (1.5 g/kg/day) not only had a preventive effect before noise exposure but also effectively reduced hearing loss when administered after exposure. The mechanism may be related to the protection of outer hair cells^119^. It is worth noting that Uemaetomari *et al.* did not observe the same effect when using other T-type blockers (such as mibefradil and flunarizine), suggesting that the efficacy of T-type calcium channel blockers may be influenced by factors such as the method and timing of administration^115^. Therefore, although calcium channel blockers have shown some promise in the prevention and treatment of NIHL, their clinical application still requires more systematic research and validation.

Although calcium channel blockers have demonstrated protective effects against noise-induced hearing loss in some experiments, their efficacy is influenced by factors such as animal models, types of drugs, and methods of administration, and the research findings are not yet consistent. Therefore, clarifying the role of calcium channels in the cochlea will help develop more targeted drugs and improve methods of administration, such as local delivery, which can enhance therapeutic efficacy. Calcium channel blockers hold great promise in the prevention and treatment of hearing loss.

### Intervention strategies for MCU

SNHL is one of the most common types of hearing impairments in clinical practice, typically resulting from damage to cochlear hair cells and their associated neurons. Recent studies have shown that mitochondrial Ca²⁺ dyshomeostasis, particularly Ca²⁺ overload mediated by the MCU, is closely associated with hair cell apoptosis and constitutes a key mechanism underlying deafness. Therefore, targeting MCU and its Ca²⁺ regulation has become a potential therapeutic target. The MCU plays a certain role in regulating excitotoxicity^120^. Its overexpression can markedly enhance the ability of mitochondria to take up Ca²⁺, leading to mitochondrial dysfunction. This, in turn, makes cells more susceptible to apoptotic signals and increases their vulnerability to cell death^121^. Under physiological conditions, mitochondrial Ca²⁺ homeostasis is primarily maintained by the coordinated actions of the MCU and NCLX.

MCU plays a crucial role in maintaining cellular Ca²⁺ homeostasis. Elevated MCU activity drives pathological mitochondrial Ca²⁺ accumulation, ultimately triggering cell apoptosis^122^, ^123^. Studies have demonstrated that after exposure to aminoglycoside antibiotics, the level of mitochondrial Ca²⁺ in dying cells significantly increases, exhibiting typical characteristics of Ca²⁺ overload^113^. Subsequently, Wang *et al.* were the first to discover that noise exposure significantly upregulates MCU expression in the OHCs of the basal turn of the mouse cochlea, while concurrently downregulating NCLX expression. This shift leads to mitochondrial Ca²⁺ overload, which may then accelerate the occurrence of NIHL by promoting the degeneration of synapses in IHCs and the loss of OHCs.

Therapeutic interventions by using siRNA-mediated gene silencing or applying the high-affinity MCU-specific inhibitor Ru360, the MCU-mediated mitochondrial calcium uptake can be effectively blocked, Ca²⁺ overload can be alleviated, and significant reduction in OHC damage as well as prevention of permanent hearing loss can be achieved^103^. Targeting MCU-mediated mitochondrial Ca²⁺ overload offers a new direction for the treatment of sensorineural hearing loss. Future research could focus on developing more efficient and safer MCU inhibitors, as well as their therapeutic potential in different types of hearing loss.

### Mitochondria-targeted treatment for hearing loss

Mitochondria-targeted therapy provides a precise intervention strategy for hearing loss by focusing on protecting cochlear hair cells and spiral ganglion neurons. These therapies aim to address mitochondrial dysfunction, minimizing oxidative damage, and reestablishing energy metabolism equilibrium. Antioxidants mainly inhibit SNHL by reducing the excessive production of ROS.

Mitochondrial ROS are by-products of cellular metabolism, a process that largely depends on Ca²⁺ signaling between the endoplasmic reticulum and mitochondria^124^. Mitochondrial Ca²⁺ can increase the production of ROS by enhancing mitochondrial activity^125^. Esterberg *et al.* demonstrated that aminoglycoside-induced hair cell death primarily occurs through ROS generation caused by mitochondrial Ca²⁺ overload. After inhibiting mitochondrial Ca²⁺ uptake with Ru360, the levels of ROS in the mitochondria and cytoplasm were significantly reduced, and the survival rate of hair cells increased. This finding provides a theoretical basis for the development of novel treatments for ototoxic hearing loss^112^. Mitochondria-targeted antioxidants have a protective effect, The mitochondria-targeted CoQ10/ubiquinone derivative MitoQ scavenges ROS by accumulating inside the mitochondria. In theory, it can reduce ototoxicity. However, studies have found that MitoQ has a limited protective effect in amikacin-induced hearing damage. It only shows partial hearing protection effects at certain high frequencies, and its direct protection of hair cells is not significant^126^. In contrast, another antioxidant, SkQR1 (a mitochondria-targeted proton-translocating cationic antioxidant), shows a more significant protective effect. Animal experiments have shown that this drug can significantly reduce the hearing loss and kidney damage caused by gentamicin, and increase the survival rate of animals, suggesting its potential in the treatment of aminoglycoside-related deafness^127^.

Noise exposure, similar to ototoxic drugs, can induce mitochondrial oxidative stress and damage. Sestrin 2 an endogenous antioxidant protein. Research indicates it protects against noise exposure by stabilizing Unc-51-like protein kinase 1 protein and activating Parkin-mediated mitophagy. Knocking out Sestrin 2 will exacerbate hair cell damage and hearing loss, suggesting that it can serve as a potential therapeutic target^128^. Mitochondria-targeted apoptosis inhibition can also suppress the progression of SNHL. Studies have found that glutamate-induced apoptosis of SGNs involves mitochondrial damage mediated by the Apoptosis-Inducing Factor. The calpain inhibitor PD150606 can effectively block this pathway, thereby reducing the cell apoptosis rate and providing a new strategy for protecting neurons^129^.

In summary, mitochondria-targeted therapy, by regulating the oxidative stress and autophagy pathways, has shown broad application prospects in the prevention and treatment of sensorineural hearing loss.

## Conclusion

SNHL is a complex multifactorial disease, and its pathogenesis is closely related to calcium homeostasis imbalance, mitochondrial dysfunction, hair cell apoptosis, oxidative stress, and other factors. Ca^2+^ is involved in activating MET channels, releasing synaptic neurotransmitters, maintaining cochlear homeostasis, and influencing auditory system integrity by regulating cell metabolism, growth, and apoptosis. Mitochondrial damage, central to cellular metabolism and Ca^2+^ regulation, can lead to ROS accumulation, and Ca^2+^ imbalance, resulting in the degeneration of hair cells and spiral ganglion neurons. In recent years, intervention strategies targeting calcium signaling pathways have been continuously expanding. The mechanisms of action of calcium channel blockers and Ca^2+^ overload mediated by MCU in sensorineural hearing loss have gradually been clarified, and initial therapeutic effects have been demonstrated in animal models. In the future, an integrated therapeutic approach combining calcium signaling regulation, mitochondrial function protection, and advanced drug delivery systems is expected to break through the limitations of traditional treatments and provide patients with SNHL with more precise, safe, and translational treatment options.

## Figures and Tables

**Table 1:** PMCA2 Gene Mutations and Associated Auditory Phenotypes in Mice and Humans

Species	Mutation/Model Name	Animal Model/Study Population	Impact on Protein Function	Hearing Phenotype	Reference
Mouse	dfw (deafwaddler)	Spontaneous mutation	Loss of Ca²⁺ pump activity	Severe congenital sensorineural hearing los	49
Mouse	dfw2j	Spontaneous mutation	Protein truncation, non-functional	Severe congenital hearing loss	53
Mouse	dfwi5	ENU-induced mutation	No PMCA2 expression	Severe hearing loss	51
Mouse	Tommy	ENU-induced mutation	Impaired ATPase function	Progressive hearing loss	55
Mouse	Oblivion (Obl)	ENU-induced mutation	Pump in non-activated state, reduced function	Obl/Obl: Severe hearing loss at birth; Obl/+: Progressive hearing loss after P20	54
Mouse	Wrigle mouse Sagami(wri)	Spontaneous mutation	Lack of PMCA2 expression in the cochlea	Severe hearing loss	50
Mouse	PMCA2-null	Targeted knockout mouse		Homozygous: Severe hearing loss; Heterozygous: Progressive hearing decline	52
Human	V586M	Families with CDH23 mutation	Reduced pump activity, exacerbated deafness	Exacerbated sensorineural hearing loss	48
